# Multiple-cluster detection test for purely temporal disease clustering: Integration of scan statistics and generalized linear models

**DOI:** 10.1371/journal.pone.0207821

**Published:** 2018-11-21

**Authors:** Kunihiko Takahashi, Hideyasu Shimadzu

**Affiliations:** 1 Department of Biostatistics, Nagoya University Graduate School of Medicine, Nagoya, Japan; 2 Department of Mathematical Sciences, Loughborough University, Loughborough, Leicestershire, United Kingdom; University of Zurich, SWITZERLAND

## Abstract

The spatial scan statistic is commonly used to detect spatial and/or temporal disease clusters in epidemiological studies. Although multiple clusters in the study space can be thus identified, current theoretical developments are mainly based on detecting a ‘single’ cluster. The standard scan statistic procedure enables the detection of multiple clusters, recursively identifying additional ‘secondary’ clusters. However, their *p*-values are calculated one at a time, as if each cluster is a primary one. Therefore, a new procedure that can accurately evaluate multiple clusters as a whole is needed. The present study focuses on purely temporal cases and then proposes a new test procedure that evaluates the *p*-value for multiple clusters, combining generalized linear models with an information criterion approach. This framework encompasses the conventional, currently widely used detection procedure as a special case. An application study adopting the new framework is presented, analysing the Japanese daily incidence of out-of-hospital cardiac arrest cases. The analysis reveals that the number of the incident increases around New Year’s Day in Japan. Further, simulation studies undertaken confirm that the proposed method possesses a consistency property that tends to select the correct number of clusters when the truth is known.

## Introduction

Whether the distribution of disease spreads randomly or clusters around particular epicenters, this has been a crucial concern in epidemiological studies. Any indication of disease clustering at an early phase of outbreak offers valuable insights into preventing us from a worse pandemic scenario or may provide us with clues to the etiology of the disease [1, 2]. A well-known monumental work would be one carried by John Snow, a British physician, in identifying the source of a cholera outbreak in Soho, London, during the middle of 19th century. He identified a water supply pump as the source of the epidemic by mapping the number of cholera victims which delineated a cluster around the pump [3].

Although the extent to which disease distributes is often discussed in a spatial context, like Snow’s map, there is parallel to purely temporal events which also attract attention from a broad public today. For example, identifying the threat of emerging infections or the risk of bioterrorist attacks is a critical role of surveillance systems, the main focus of which is to monitor the incidence or prevalence of specific health problems over time within a well-defined population [4].

To identify meaningful clusters, in other words, to investigate a regional or temporal tendency in the presence of certain diseases, whether the disease risk is relatively high to other surrounding regions or subsequent time periods, a number of statistical tests have been proposed and are widely used [5]. These tests are classified based on their purpose. For example, focused tests have been developed to detect the existence of a local cluster around a predetermined point source, while general tests search for clusters without any preconceived assumptions about their location [6]. The general test framework further consists of two types of tests. Global clustering tests, such as those developed by Moran [7], Whitemore *et al* [8], Oden [9], Tango [10], Rogerson [11] and Bonetti and Pagano [12], detect the presence of clusters in a study area without determining the statistical significance of individual clusters. On the other hand, cluster detection tests (CDTs), such as those proposed by Besag and Newell [6], Turnbull *et al*. [13], Kulldorff and Nagarwalla [14], Kulldorff [15, 16], Tango [17, 18], Duczmal and Assunção [19], Tango and Takahashi [20, 21], Takahashi *et al*. [22], and Jung [23], possess the flexibility to accommodate spatial, temporal, or spatio-temporal data, and enables the determination of whether a disease pattern is completely random over the space of study without prior information while indicating regions or time periods with high disease prevalence.

The scan statistic is one of the most powerful elements of the CDT since it is based on a concrete statistical framework—i.e., the maximum likelihood ratio; examples include the circular scan statistic [15] along with the SaTScan software [24] and the flexibly shaped scan statistic [20] implemented in the FleXScan software [25]. These have widely been applied to important challenges in various fields such as the epidemiology of cancer and other diseases, infectious disease surveillance, parasitology, psychology, ambulance logistics, medical imaging, genome-wide association analyses, and drug and vaccine development [24, 26–28].

A shortcoming of these approaches is, however, the fact that most of them focus on ‘single’ cluster detection while investigating the extended study space or period within which more than one cluster is expected. To detect more than one cluster, the ordinary scan statistic procedure, including the circular and flexibly shaped ones, is iteratively applied after the identification of the first (primary) cluster; additional, mutually exclusive ‘secondary’ clusters are then sequentially detected by the likelihood ratio statistic—we hereafter refer to this conventional procedure as the secondary-cluster procedure. The procedure can only evaluate these clusters one by one, and each corresponding *p*-value is calculated as if the cluster were the primary one. This fact indicates that the current approach does not provide an accurate assessment of the selected multiple clusters.

In the present work, we construct a general test procedure that enables the simultaneous evaluation of multiple clusters, focusing on a purely temporal Poisson model. Combining generalized linear models (GLMs) and the ordinary scan statistic procedure, the new testing framework stands directly on the full-likelihood principle that can easily be amalgamated with an information criterion approach to select clusters via GLMs. This procedure becomes, as will be described in a later section, a natural extension of scan statistic—i.e., the conventional secondary-cluster procedure, and can accurately evaluate multiple clusters as a whole. An application study adopting the proposed procedure is then discussed in the context of a real-world example—i.e., temporal data on the daily incidence of out-of-hospital cardiac arrest cases in Japan [28]. The results are compared to those obtained by the secondary-cluster procedure. The consistency property of the proposed procedure, a desirable property, whether it tends to select the correct number of clusters is also examined via simulations studies.

## Methods

In this section, we first present an overview of the ordinary scan statistic framework implemented in SaTScan and FleXScan for a single-cluster detection and then describe the conventional secondary-cluster procedure for the multiple-cluster detection proposed by Kulldorff [15] using a Poisson GLM as described in Jung [23]. We then propose a new test procedure that simultaneously evaluates multiple clusters using both GLMs and an information approach. We also show that the new procedure encompasses the secondary-cluster procedure as a special case.

### Ordinary scan statistic for single-cluster detection and secondary-cluster procedure

A study space (area or time period) *G* consists of *m* segments, each of which corresponds to the smallest element of the space; this can be a county, month, or day depending on the type of data. The number of cases within segment *i*—i.e., *Y*_*i*_—is presumed to follow an independent Poisson distribution, with an expected value *μ*_*i*_—i.e., *Y*_*i*_|*μ*_*i*_ ∼ Poisson(*μ*_*i*_), which is henceforth denoted in lowercase as *y*_*i*_, *i* = 1, 2, …, *m*. Let W denote the set of all potential scanning zones (the sets of connected segments) of any size, construction of which relies on an employed scanning method. Assuming the window *w* (∈ W) as a hot-spot cluster in which the number of cases, *y*_*i*_, is higher than in other parts of the study space, the expected number of cases can be modelled as

$$\begin{array}{c} \text{log}\;\mu_{i}=\text{log}\left(\theta_{i}\mu_{i}^{0}\right)=\alpha +\beta z_{i}+\text{log}\;\mu_{i}^{0}, \end{array}$$ (1)

with the indicator variable *z*_*i*_ = 1 if *i* ∈ *w* and *z*_*i*_ = 0 otherwise (*i* ∉ *w*). For those segments that fall into a hot-spot cluster (*w*), a parameter of model (1) becomes *θ*_*i*_ = *θ*_*w*_ = exp(*α* + *β*) for *β* ≥ 0. On the other hand, for those that fall outside the cluster ($\bar{w}$), the parameter is $\theta_{i}=\theta_{\bar{w}}=\text{exp}(\alpha )$. The constant term $\mu_{i}^{0}≔\mu_{i}^{0}\left(x_{i}\right)$ is often modelled as a function of other covariates ***x**_i_*, such as demographic or environmental factors; this yields the null model—i.e., the expected number of cases if there is no cluster in the study space such that *β* = 0. The null model is therefore $\text{log}\;\mu_{i}=\alpha^{0}+\text{log}\;\mu_{i}^{0}$.

The likelihood function of model (1) is generated as follows: $f_{i}\left(y_{i}|z,\psi \right)=f(y_{i}|\mu_{i}^{0},z,\psi )$ is the probability function of *Y*_*i*_ = *y*_*i*_ given the location of hot-spot window, ***z*** ≔ ***z***(*w*) = (*z*_1_, *z*_2_, …, *z*_*m*_), and the parameters ***ψ*** = (*α*, *β*) and $\mu_{i}^{0}$. The probability function $f(y_{i}|\mu_{i}^{0},z,\psi )$ can be expressed as either $f(y_{i}|\mu_{i}^{0},z,\theta_{w})$ for *i* ∈ *w* or $f(y_{i}|\mu_{i}^{0},z,\theta_{\bar{w}})$ for $i\in \bar{w}$, $w\cap \bar{w}=\emptyset$. Hence, the conditional log likelihood function is $l(\psi |z)≔\text{log}\;\{\prod_{i=1}^{m}f_{i}\left(y_{i}|z,\psi \right)\}=\text{log}\;[\prod_{i=1}^{m}{\left\{f(y_{i}|\mu_{i}^{0},z,\theta_{w})\right\}}^{z_{i}}{\left\{f(y_{i}|\mu_{i}^{0},z,\theta_{\bar{w}})\right\}}^{1-z_{i}}]$. This leads to the following statistical hypothesis test to detect a single cluster:

$$\begin{array}{c} H_{0}:\theta_{w}=\theta_{\bar{w}}\;\text{for}\;\forall w\in W;\;H_{1}:\theta_{w}>\theta_{\bar{w}}\;\text{for}\;\exists w\in W. \end{array}$$

The null hypothesis *H*_0_ states that there is no cluster—i.e., ***z*** = **0**, whereas the alternative hypothesis *H*_1_ asserts that there is a hot-spot window *w*—namely, ***z*** = ***z***(*w*)—in which the underlying disease risk is higher than in the other parts of the study space. It is important to note that the two hypotheses can also be expressed in equivalent forms with respect to *β* as *H*_0_: *β* = 0 for ∀*w* and *H*_1_: *β* > 0 for ∃*w*.

The maximum log likelihood ratio (LLR) for a given *w* is

$$\begin{array}{c} LLR\left(w\right)=l(\psi =(\alpha ,\beta )|z=z(w))-l(\psi =(\alpha^{0})|z=0) \end{array}$$ (2)

and the conventional scan statistic is defined as $\max_{w\in W}LLR\left(w\right)$. A window w* that attains $\max_{w\in W}LLR\left(w\right)$—i.e., $\max_{w\in W}\;l(\psi |z=z(w))$—, is identified as the most likely (primary) cluster. The Monte Carlo method is commonly used to evaluate statistical significance. A large number of random datasets are generated under the null hypothesis, and the test statistic is then calculated for each dataset; the upper or lower percentiles provide a Monte Carlo-based *p*-value.

The described procedure above was intended to identify only the primary cluster, $w_{1}^{*}=w^{*}$. For multiple cluster detection, Kulldorff [15] extended its use; the procedure was repeatedly used to identify other clusters, namely ‘secondary’ clusters, $w_{2}^{*}$, $w_{3}^{*}$, …, among which there were no overlaps—i.e., $w_{k}^{*}\cap w_{k^{\prime }}^{*}=\emptyset$ for *k* ≠ *k*′ and consequently, their LLRs always followed a descending order, $LLR(w_{1}^{*})>LLR(w_{2}^{*})>\cdots >LLR(w_{k}^{*})>\cdots$. The statistical significance of secondary clusters was evaluated in the same way as that of the most likely cluster; i.e., the log-likelihood ratio of each secondary cluster was compared to the maximum log-likelihood ratio calculated from randomly generated data sets. This procedure is simple and easy to use, although it is limited by the fact that it only evaluates clusters individually as if each one were the primary cluster, and does not assess multiple clusters as a whole.

### A new framework for multiple-cluster detection test procedure

To construct a new procedure, we first show here that the multi-cluster model can be formulated in the form of mixture Poisson GLMs and then derive the likelihood function. This new formulation recognizes the cluster selection problem as a model selection problem for which we can embrace the information criterion paradigm. To choose an appropriate number of clusters, we then propose a new criterion delivered from the likelihood function in the same manner as BIC. The computational aspect of the proposed methods is reasonably straightforward utilizing existing statistical software although, some technical details are explained at the end of this section.

#### Multiple cluster model and its likelihood

Assuming that there are *K* clusters [***w*** = (*w*_1_, *w*_2_, …, *w_K_*)] in a space *G*, each mutually exclusive window *w*_*k*_ contains a set of adjacent segments as a cluster—i.e., $w_{k}\cap w_{k^{\prime }}=\emptyset$ for *k* ≠ *k*′. Note that *K* = 0 and *K* = 1 indicate that there is no cluster and a single cluster in the study space, respectively. The expected number of cases *μ*_*i*_ of segment *i* can be modelled as an extension of (1) as follows:

$$\begin{array}{c} \text{log}\;\mu_{i}=\text{log}\left(\theta_{i}\mu_{i}^{0}\right)=\alpha +\sum_{k=1}^{K}\beta_{k}z_{ki}+\text{log}\;\mu_{i}^{0} \end{array}$$ (3)

for *K* ≥ 1 and $\text{log}\;\mu_{i}=\alpha^{0}+\text{log}\;\mu_{i}^{0}$ for *K* = 0. Here, the indicator variable *z*_*ki*_ = 1 if *i* ∈ *w*_*k*_ and *z*_*ki*_ = 0 otherwise. Note that *β*_*k*_ > 0.

Recalling the notation in the previous section, $f(y_{i}|\mu_{i}^{0},z,\psi )$ is the probability function of *Y*_*i*_ = *y*_*i*_ given ***z*** ≔ ***z***(*w*) = (*z*_*ki*_)—which is now a *K* × *m* matrix—and the parameters ***ψ*** = (*α*, *β*_1_, *β*_2_, …, *β_K_*). The conditional log likelihood function can be written as $l\left(\psi |z\right)≔\text{log}\;[\prod_{i=1}^{m}\prod_{k=0}^{K}{\left\{f(y_{i}|\mu_{i}^{0},z,\psi )\right\}}^{z_{ki}}]$, where *z*_0*i*_ = 1 if $i\notin \cup_{k=1}^{K}w_{k}$, and *z*_0*i*_ = 0 otherwise. We assume ***z*** to be randomly selected in accordance with a probability function *h*(***z***). The complete (full) log likelihood function of ***ψ*** is then expressed as:

$$\begin{array}{c} l\left(\psi \right)=\text{log}\;L\left(\psi \right)=\text{log}\;[\prod_{i=1}^{m}\prod_{k=0}^{K}{\left\{f(y_{i},z_{ki}|\mu_{i}^{0},\psi )\right\}}^{z_{ki}}]=l\left(\psi |z\right)+\text{log}\;\{h(z)\} \end{array}$$

where *L*(***ψ***) is the likelihood function of ***ψ***. The maximum log likelihood ratio statistic *LLR*(*w*) (2) is equivalent to the maximum of *l*(***ψ***) for *K* = 1 with *h*(***z***), which assumes a constant probability under the alternative hypothesis *H*_1_.

#### Candidates of multiple clusters *w*

The multiple-cluster model (3) depends on the choice of ***z***—i.e., ***w*** among a large number of combinations of sets in W. Although the choice is, indeed, a search problem in a general context, we here focus on extending the secondary-cluster procedure due to its natural interpretation. We are interested in evaluating whether each of candidate clusters is able to reject the null hypothesis on its own strength, significance in other words, in terms of the likelihood statistic value. In some sense, it is like a regression analysis where each variable is entered in a separate regression model and then evaluated without adjusting for other variables [29]. Thus, we adopt the conventional scan statistic approach to select candidate clusters. First, the primary cluster $w_{1}^{*}$ is selected as candidate *w*_1_ in ***w***; then, with a pre-defined maximum number of candidates, among which there are no overlaps—i.e., *K*_*max*_ (≥ 1)—(*K*_*max*_ − 1) secondary clusters, $w_{2}^{*},w_{3}^{*},\ldots ,w_{K_{max}}^{*}$, are selected one by one, regardless of their significance. In practice, we predefine the maximum number of candidates (e.g., *K*_*max*_ = 10, 20, …) or a P-value threshold, *p*_*s*_, (e.g., *p*_*s*_ < 0.5, 0.8, 1.0). It should be noted that *K*_*max*_ = 1 corresponds to detection of a single cluster using the conventional scan statistic procedure. Candidate selection may differ depending on the scanning method that is adopted (e.g., circular, flexible, etc.).

#### Selecting an appropriate *K* and significance test

To select an appropriate number of clusters, *K*(≤ *K*_*max*_), we propose a new information criterion approach that chooses *K* in favor of the largest marginal likelihood, *ML*(***y***, ***z***) = ∫ exp {log *L*(***ψ***)} *g*(***ψ***)*d**ψ***, where *g*(***ψ***) is a prior probability function of parameter ***ψ***. This can be achieved by the same manner in deriving the Bayesian information criterion (BIC). Applying the Taylor expansion and Laplace approximations to the integral above [30], the log marginal likelihood is approximated as

$$\begin{array}{ccc} -2\;\text{log}\;ML(y,z) & \approx & -2\sum_{i=1}^{m}\sum_{k=0}^{K}z_{ki}\{\text{log}\;f(y_{i}|\mu_{i}^{0},z,\hat{\psi })\}-2\;\text{log}\;(h(z))\\ & & \;+q\;\text{log}\;m+\text{log}\;|J\left(\hat{\psi }\right)|-q\;\text{log}\left(2\pi \right)-2\;\text{log}\left(g\left(\hat{\psi }\right)\right) \end{array}$$

where $\hat{\psi }$ is the maximum likelihood estimator of ***ψ***,

$$\begin{array}{c} J\left(\hat{\psi }\right)=-\frac{1}{m}\frac{\partial^{2}l\left(\psi |z\right)}{\partial \psi \partial \psi^{\prime }}{|}_{\psi =\hat{\psi }} \end{array}$$

and *q* = *K* + 1. The model evaluation criterion can then be obtained by omitting terms with an order less than *O*(1) with respect to the large sample size *m*; that is,

$$\begin{array}{c} C\left(K\right)=-2l\left(\hat{\psi }|z\right)-2\;\text{log}\left(h\left(z\right)\right)+\left(K+1\right)\;\text{log}\;m,\;\left(K\geq 1\right). \end{array}$$ (4)

To select an appropriate number of clusters, *K*, we define a relative difference statistic based on the criterion *C*(*K*) as

$$\begin{array}{c} RDC\left(K\right)=\left(C_{0}-C\left(K\right)\right)/C_{0}, \end{array}$$

where *C*_0_ = *C*(0), the criterion under the null model. Appropriate multiple clusters are selected from the set of candidates $\tilde{w}=\left(w_{1},w_{2},\ldots ,w_{K}\right)$ with respect to max_*K*_ *RDC*(*K*). It should be noted that there is a clear link to the conventional cluster detection approach: the candidate cluster $\tilde{w}$ attains max_*w*_ *LLR*(*w*) if *K* = 1 under *K*_*max*_ = 1.

To calculate the proposed criterion (4), the probability function *h*(***z***) needs to be specified; for purely temporal cases, we recommend to use *h*(***z***) = (1/*m*)^*K*^ as an approximation of the probability of selecting locations ***w*** when the window size is relatively very small, #{*i*|*i* ∈ ***w***} ≪ *m*, with respect to the whole data size *m*. Hence, a temporal cluster selection criterion is given as

$$\begin{array}{c} C\left(K\right)=-2l\left(\hat{\psi }|z\right)+\left(3K+1\right)\;\text{log}\;m\;\;\left(K\geq 1\right). \end{array}$$

The statistical significance of appropriate models is evaluated by the Monte Carlo hypothesis testing procedure in the same manner as with the standard scan statistic. Under the null hypothesis, a large number of random datasets are generated; however, for each of these, max_*K*_ *RDC*(*K*) is instead calculated as a test statistic.

#### Computation

All the numerical computation can easily be carried out by R [31] using the glm function that fits model (3) and then calculates the log-likelihood ratios. The Monte Carlo-based *p*-value [32] is computed in the following way. First, we calculate the observed test statistic based on the observed data set. Second, we compute the same statistic for a large number *N*_*rep*_ of data sets which are simulated independently under the null hypothesis of no clustering. The simulated *p*-value is obtained from the rank of the statistic among *N*_*rep*_ + 1 values of the statistic. If this rank is *R*, then *p*-value = *R*/(*N*_*rep*_ + 1). The Monte Carlo simulated *p*-value is not necessary the same value all the time due to the randomly simulated data. In other words, another independent set of *N*_*rep*_ realisations will result in a slightly different *p*-value. However, a larger number of replications *N*_*rep*_ consequently provides a more stable *p*-value. The number of replication *N*_*rep*_ is usually set as 999 or 9,999.

## Application and simulation data

To illustrate how the proposed procedure performs in detecting temporal multiple-clusters, we apply it to real-world data, the daily out-of-hospital cardiac arrest (OHCA) cases in Japan and compare the results with those obtained from the conventional secondary-cluster procedure. Further, the consistency of the proposed procedure is also investigated via a simulation study. The aspect of consistency, whether the proposed procedure tends to select the correct number of clusters when the truth is known, is an essential part in evaluating the performance of the proposed framework and the desired property as a reliable procedure.

### An application: Detecting temporal clusters in daily incidence of out-of-hospital cardiac arrest cases

As an example, the Japanese OHCA data for the period of 2005–2011 (2,260 days) were studied and a total of 701,651 cases (MNC; male con-cardiac cases) were analyzed following a previous work [28]. The expected number of OHCA cases $\mu_{i}^{0}$ on day *i* in model (3)—that is, the null expectation with no cluster—was estimated by a Poisson regression model for the observed OHCA cases *y*_*i*_, while accommodating the following five factors into the model: year, month, day of the week, holidays, and temperature, in two-by-two stratification by sex (male/female) and the etiology of arrest (cardiac/non-cardiac). For more details see Takahashi and Shimadzu [28].

To determine whether OHCA incidence showed specific temporal clustering patterns, we calculated the scan statistic implemented with the restricted likelihood ratio [18, 21], which was an improved version of Kulldorff’s standard scan statistic [15]. We set two default arguments of the program: the maximum temporal length of a cluster was 20 days and the pre-specified significance level for a restriction was *α*_1_ = 0.2. The significance level of the test was set as 0.05, and the *p*-value was calculated from 999 replications of the Monte Carlo hypothesis test.

#### Data and data accessibility

Daily ambulance records of OHCA cases were obtained from the All-Japan Utstein registry data of cardiopulmonary arrest patients provided by the Fire and Disaster Management Agency (FDMA). This was a nationwide and population-based registry system of OHCA cases available since 2005, in accordance with the Utstein guidelines. Since all the records were made anonymous by FDMA, according to the informed consent guidelines in Japan, we were exempt from obtaining informed consent from each patient to use this dataset. All the data set used in the present study is public data and available from the Ministry of Internal Affairs and Communications, Japan upon request at fdma-goiken@ml.soumu.go.jp, referring to the All-Japan Utstein registry data 2005–2011.

### Simulation studies

We carried out simulation studies to assess the performance of the proposed framework in detecting multiple clusters from time-series data. The purpose of it is to check if the proposed framework possesses a consistent property that tends to select the correct number of clusters when the truth is known. Here, the expected count of daily OHCA MNC cases from the null hypothesis served as an example time series. We assumed six periods [A–F; Table 1], during which the incidence of OCHA cases—that is, the relative risk (RR) of OCHA—was set at a high value, and we introduced seven scenarios with different RR for each of the periods [Table 2]. For example, Scenario S2.1 had three cluster periods (A–C) for which the RR was set as 2.0; for the remainder of the time series, including periods D–F, the RR was 1.0, indicating that there was no cluster. We generated 1000 datasets for each scenario and compared the estimated power calculated from the two cluster detection tests with the significance level of 0.05.

**Table 1:** Assumed cluster periods with expected counts under the null model in simulation studies.

	period	# days	expected counts
A	2006/01/01–03	3	(115.10, 131.43, 122.34)
B	2005/01/01–03	3	(103.08, 108.53, 124.18)
C	2005/04/01–03	3	(76.44, 77.10, 84.35)
D	2005/02/01	1	(87.27)
E	2007/01/01–05	5	(127.77, 117.05, 114.54, 99.36, 100.34)
F	2007/04/01–05	5	(84.83, 81.85, 78.47, 79.52, 82.61)

**Table 2:** Assumed relative risks in simulation studies.

scenario	# clusters	# days	Relative Risk (RR)*					
			A	B	C	D	E	F
S0	0	0						
S1	1	3	1.5					
S2.1	3	9	1.2	1.2	1.2			
S2.2	3	9	1.3	1.3	1.3			
S2.3	3	9	1.5	1.5	1.5			
S2.4	3	9	2.0	2.0	2.0			
S3.1	6	20	2.0	2.0	2.0	2.0	2.0	2.0
S3.2	6	20	1.3	1.5	2.0	2.0	1.3	2.0

* All the blanks should read as Relative Risk (RR) is 1.

## Results

### An application: Detecting temporal clusters in daily incidence of out-of-hospital cardiac arrest cases

**Fig 1:**
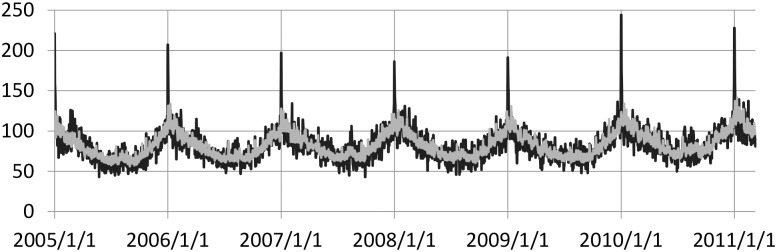
The black solid line represents the daily out-of-hospital cardiac arrest (OHCA), male non-cardiac cases (MNC), in Japan from 1 January 2005 to 10 March 2011 (*m* = 2, 260 days); total 185,819 cases, and maximum and minimum of daily counts were 244 and 43, respectively. Grey line overlays the null expected counts (Takahashi and Shimadzu [28]).

Fig 1 shows the number of observed cases *y*_*i*_ and the expected counts $\mu_{i}^{0}$ under the null hypothesis for male non-cardiac cases (MNC) with 185,819 cases.

The conventional secondary-cluster procedure first detected the most likely cluster of 2-day length and then seven other secondary clusters (Table 3). It should be noted that since random numbers were used in the Monte Carlo procedure, our *p*-values slightly differed from those previously reported [28].

**Table 3:** Detected significant temporal clusters in daily incidence of out-of-hospital cardiac arrest (OHCA), male non-cardiac cases (MNC), by the secondary-cluster procedure.

rank	cluster	clustered period	cases	expects	RR	*p*-value *p*_*s*_
1	*w*_1_	2010/01/01—2010/01/02	423	232.43	1.82	0.001
2	*w*_2_	2005/01/01—2005/01/02	381	211.61	1.80	0.001
3	*w*_3_	2011/01/01	228	111.40	2.05	0.001
4	*w*_4_	2008/12/31—2009/01/03	630	444.54	1.42	0.001
5	*w*_5_	2006/01/01	207	115.10	1.80	0.001
6	*w*_6_	2008/01/01—2008/01/04	614	446.72	1.37	0.001
7	*w*_7_	2007/01/01—2007/01/02	344	244.83	1.41	0.001
8	*w*_8_	2011/01/02—2011/01/06	711	589.98	1.21	0.011

RR: relative risk

We then applied our proposed framework to the same data. Candidate clusters were selected with a threshold *p*-value *p*_*s*_ < 1.0, which was equivalent to setting *K*_*max*_ = 25 for the observed data. The suggested multiple cluster model had eight clusters, *K* = 8 [*C*_0_ = 17433.89, *C* = *C*(8) = 17041.46, *RDC*(8) = 0.02251].

**Fig 2:**
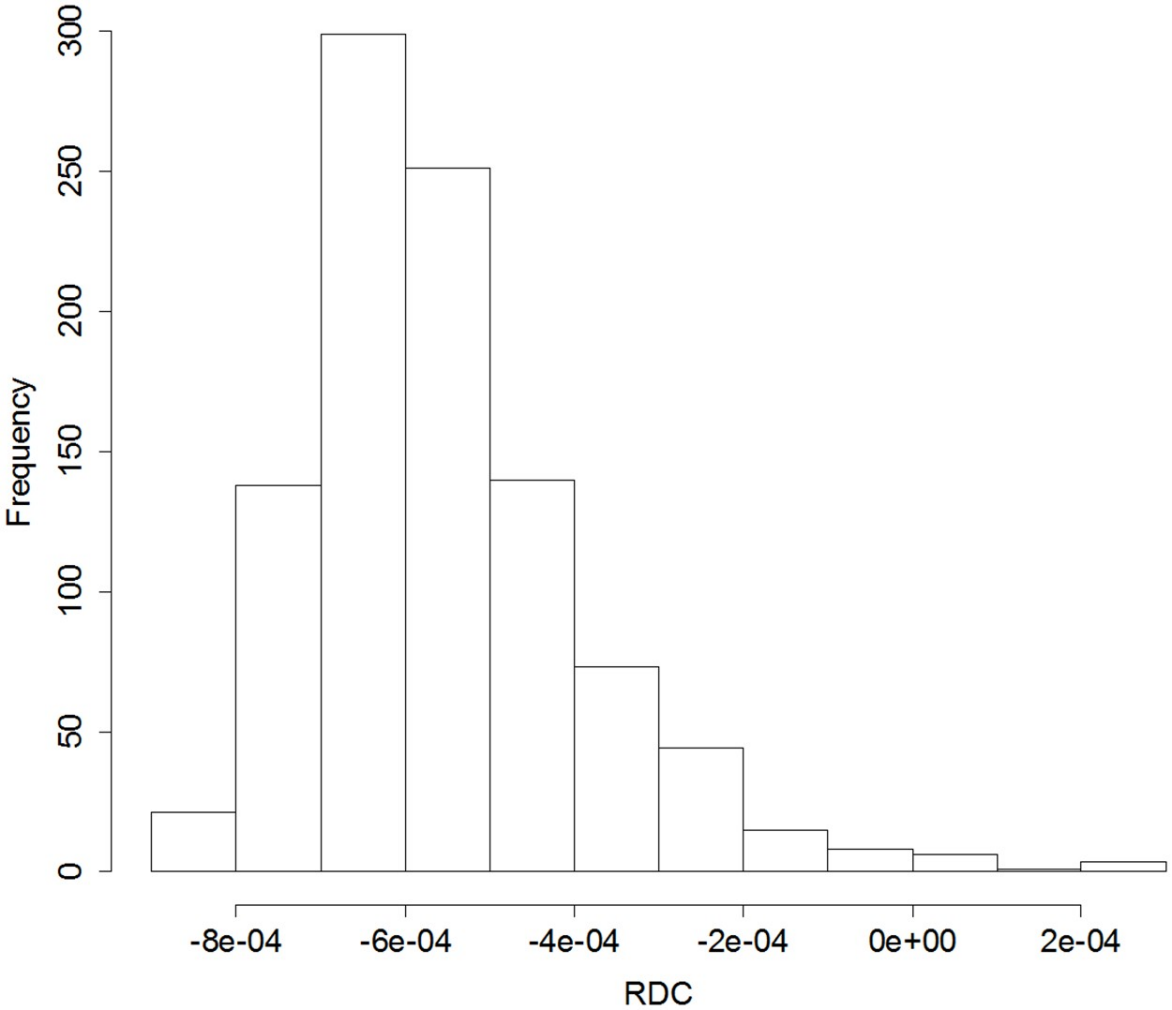
Histogram of the null distribution of *RDC*(*K*).

Fig 2 shows the null distribution of *RDC*(*K*) generated by the Monte Carlo-simulated data with 999 replications; this was similar to the results obtained by the secondary-cluster procedure. However, the proposed procedure calculated a *p*-value of 0.001 for the multiple-cluster model from the upper fifth percentile of the statistic *RDC*_5%_ = −0.00025. The selected multiple-cluster model by the proposed procedure is shown in Table 4.

**Table 4:** Detected multiple-cluster model by the proposed procedure.

	clustered period	coef.	OR	95%CI	*p*-value
intercept		−0.006			0.0077
*w*_1_	2010/01/01—2010/01/02	0.605	1.831	(1.662, 2.012)	< 0.0001
*w*_2_	2005/01/01—2005/01/02	0.594	1.812	(1.636, 2.000)	< 0.0001
*w*_3_	2011/01/01	0.722	2.059	(1.803, 2.339)	< 0.0001
*w*_4_	2008/12/31—2009/01/03	0.355	1.426	(1.317, 1.541)	< 0.0001
*w*_5_	2006/01/01	0.593	1.810	(1.574, 2.068)	< 0.0001
*w*_6_	2008/01/01—2008/01/04	0.324	1.383	(1.276, 1.500)	< 0.0001
*w*_7_	2007/01/01—2007/01/02	0.346	1.414	(1.270, 1.569)	< 0.0001
*w*_8_	2011/01/02—2011/01/06	0.193	1.213	(1.126, 1.304)	< 0.0001

coef.: estimated coefficients; OR: odds ratio; 95%CI: its 95% confidence interval; and *p*−value: the *p*-value of the estimated coefficient.

**Fig 3:**
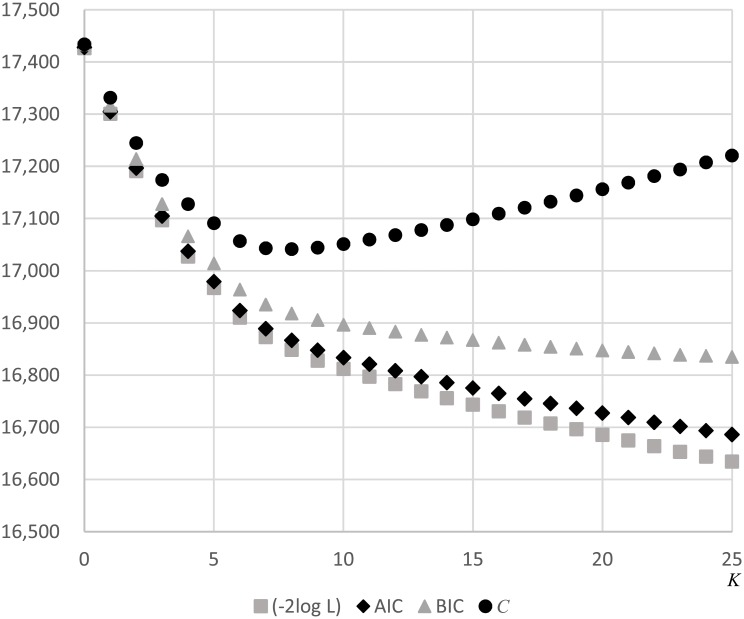
Values of the proposed criterion *C* for each *K*, with other criteria, −2log likelihood (−2log L), AIC and BIC.

Fig 3 shows the value of the proposed criterion *C* for each *K* calculated from the observed data along with other criteria such as −2log likelihood, Akaike information criterion (AIC), and Bayesian information criterion (BIC). Our criterion *C* reached the minimum value—i.e., max_*K*_ *RDC*(*K*)—at *K* = 8, unlike the other criteria that monotonically decreased with an inflection point at around *K* = 8.

Takahashi and Shimadzu [28] also investigated temporal clusters for other three groups such as male cardiac (MC) with 224,661 cases, female non-cardiac (FNC) with 128,491 cases, and female cardiac (FC) with 162,680 cases. Using the conventional secondary-cluster procedure, nine clusters (MC and FNC) and seven clusters (FC) were identified at the significance level *p*_*s*_ < 0.05 based on max*LLR*(*w*). On the other hand, our proposed procedure yielded six clusters (MC and FNC) and five clusters (FC), both with *p*-value of 0.001. Most of the periods excluded by the proposed procedure among the secondary clusters of the conventional procedure were not around New Year’s Day with *p*_*s*_ > 0.01.

### Simulation studies

We generated 1,000 datasets for each scenario and compared the estimated power calculated from the two cluster detection tests with the significance level 0.05. Table 5 shows the total power, which was calculated as the sum of rejection rates over the number of clusters with *K* > 0. We calculated the sensitivity (Sen) and positive predictive value (PPV) of days detected as significant, as well as their averages and the number of detection with Sen = 1 and PPV = 1 among 1, 000 sets.

**Table 5:** The power of the secondary-cluster and the proposed procedures in the simulation study, along with the sensitivity (Sen) and the positive predictive value (PPV) of days detected significantly.

	N.S. (*K* = 0)	*K* = 1	*K* = 2	*K* = 3	*K* = 4	*K* = 5	*K* = 6	*K* = 7	total power	Sen (avg*)	Sen = 1	PPV (avg*)	PPV = 1
S0: null RR = 1.0													
s-c proc.	0.951	0.049							0.049	—	—	—	—
proposed proc.	0.951	0.049							0.049	—	—	—	—
S1: one cluster (three days) RR = 1.5													
s-c proc.		0.959	0.040	0.001					1.000	0.996	0.988	0.971	0.919
proposed proc.		0.994	0.006						1.000	0.996	0.988	0.986	0.954
S2.1: three clusters (nine days) RR = 1.2													
s-c proc.	0.430	0.436	0.123	0.011					0.570	0.361	0.003	0.870	0.376
proposed proc.	0.426	0.531	0.043						0.574	0.313	0.000	0.878	0.405
S2.2: three clusters (nine days) RR = 1.3													
s-c proc.	0.009	0.118	0.455	0.406	0.012				0.991	0.728	0.266	0.935	0.658
proposed proc.	0.009	0.315	0.469	0.206	0.001				0.991	0.605	0.140	0.946	0.734
S2.3: three clusters (nine days) RR = 1.5													
s-c proc.			0.004	0.962	0.034				1.000	0.992	0.940	0.978	0.847
proposed proc.			0.011	0.984	0.005				1.000	0.990	0.934	0.984	0.872
S2.4: three clusters (nine days) RR = 2.0													
s-c proc.				0.960	0.039	0.001			1.000	1.000	1.000	0.991	0.957
proposed proc.				0.990	0.010				1.000	1.000	1.000	0.997	0.987
S3.1: six clusters (20 days) RR = 2.0													
s-c proc.							0.976	0.024	1.000	0.999	0.999	0.997	0.972
proposed proc.						0.002	0.989	0.009	1.000	0.999	0.997	0.998	0.987
S3.2: six clusters (20 days) RR = 1.3, 1.5, 2.0													
s-c proc.					0.003	0.170	0.795	0.032	1.000	0.952	0.597	0.976	0.669
proposed proc.					0.012	0.276	0.704	0.008	1.000	0.934	0.526	0.979	0.699

* averages among the custers detected as *K* > 0; Sen = 1: #{*Sen* = 1}/1000; PPV = 1: #{*PPV* = 1}/1000; s-c proc.: secondary-cluster procedure.

For the null scenario S0, the total power—i.e. probability of type I error—was 0.049 for both procedures and was very close to the significance level 0.05. In the single-cluster scenario [S1 (RR = 1.5)], both procedures had a total power equal to 1.0 but ours performed slightly better in the detection of a single cluster (*K* = 1). The total power for the three cluster scenarios [S2 (i)–(iv)] varied depending on RR. The power was lower—almost 0.57—for RR = 1.2, and increased to 0.991 for RR = 1.3. The total power for detection of *K* = 3 was also low for RR = 1.2 and 1.3; notably, they were lower for the proposed procedure as compared to the secondary-cluster procedure. On the contrary, the proposed procedure performed better in detecting *K* = 3 for RR = 1.5 and 2.0. Lastly, for the scenario with six clusters (S3), the total power was 1.0 for both procedures and all scenarios. The detection power of *K* = 6 clusters by the proposed procedure was slightly higher for scenario (i) with RRs of 2.0, while the secondary-cluster procedure showed higher power for scenario (ii) with different RRs. As such, the total power was almost the same for both procedures, and the detection power in terms of selecting the correct number of multiple clusters varied according to the scenario. The sensitivity of the secondary-cluster procedure was slightly higher than that of the proposed procedure, although the latter performed better in terms of PPV and the number detected with PPV = 1, suggesting that the secondary-cluster procedure tends to detect additional clusters.

## Discussion

We have proposed a general test procedure that enables the simultaneous evaluation of multiple clusters as an extension of the conventional secondary-cluster procedure, focusing on a purely temporal Poisson model. The Japanese OHCA data analysis has highlighted the most advantageous aspect of the new procedure, that is, it can evaluate the *p*-value for the whole multiple clusters, as opposed to the secondary-cluster procedure which cannot. This aspect becomes a key to the detection of meaningful multiple clusters. Our procedure has detected the equal or less number of clustered periods in the OHCA data, compared to ones of the secondary-cluster procedure [28]. This may seem to be a subtle difference although, it requires more careful interpretation regarding the *p*-value reported by Monte Carlo simulated data. Note that the individual *p*-values, *p*_*s*_, of the clustered periods commonly identified by both of the procedures state high significance with *p*_*s*_ = 0.001. On the other hand, for those clusters being excluded by the proposed procedure indicate relatively large *p*-values. It is not difficult to imagine that such *p*-values may vary due to Monte Carlo simulated data with 999 replications. This fact clearly suggests that our proposed procedure seems to be more robust and is capable of detecting the genuinely significant clusters, which is not affected by the simulated random data in this application.

Several studies have reported the detection of multiple clusters using scan statistics. In one study proposing an adjusted *p*-value, a sequential approach is used [29]. Although it performs with higher power than the conventional approach, the relative sizes of adjusted *p*-value for secondary clusters are irrelevant to the order in which the clusters are sequentially detected; that is, the *k*-th cluster may have a smaller *p*-value than the previously detected (*k* − 1)-th cluster. Moreover, this procedure can only evaluate the significance of individual clusters but not of multiple clusters as a whole.

In the spatial context, a multiple-cluster detection procedure using spatial scan statistics has also been proposed [33, 34]. However, these methods are also unable to assess the significance of multiple clusters as a whole. As a similar approach to us in terms of adopting a model selection procedure, Zhang et al. [35] have developed a generalized linear mixed model with Moran’s *I* statistic and utilised a stepwise procedure that allows multiple-cluster evaluation, accounting for random spatial effects. They have, however, concluded that the power is lower than that of the standard scan statistic [35]. A recent study [36] proposed a multiple-cluster detection procedure in the spatial context, adopting the quasi-likelihood approach that deals with spatial correlation. One drawback is that quasi-likelihood is not able to produce the likelihood or information criterion. This fact indicates that the quasi-likelihood approach still suffers from the multiple testing problem, whereas our approach avoids the issue by utilising the model selection framework with the proposed information criterion that stands on the full-likelihood principle. We leave it for future work to undertake detail comparisons of these quasi- and full-likelihood approaches in the multiple-cluster detection context.

We have proposed an information criterion procedure to select an appropriate number of clusters for detection. This approach has been used for model selection in more general statistical modelling contexts—for instance, to estimate the number of multiple clusters [37, 38] and finite mixture [39] models, for which traditional AIC and BIC are used. However, in specific situations with large datasets, conventional information criteria including −2log likelihood, AIC, and BIC were unable to accurately select an appropriate number of clusters and performed poorly (Fig 3). The proposed criterion was derived from the marginal likelihood of the multiple-cluster model (3) and accounted for the probability distribution of selected candidate clusters. Our examples and simulations clearly demonstrate that the proposed criteria performed well in terms of identifying ``true'' multiple-clusters assumed in simulation studies as an appropriate multiple-cluster model.

A more conservative *p*-value is calculated by the secondary-cluster procedure as compared to the primary cluster procedure [29, 40]. Consequently, the former identifies fewer significant secondary clusters relative to true clusters. However, this was not the case in our simulation study, which showed that the secondary-cluster procedure detected extra clusters. Our example and simulation study demonstrated that the proposed framework performs well, although there are outstanding challenges to be addressed by future work. Firstly, multiple-cluster detection depends on the scanning method that is initially used; we adopted the conventional secondary-cluster procedure to pre-select candidates for a GLM. Therefore, choosing the optimal scan statistic with high detection accuracy is essential. Secondly, our illustration and simulation were carried out only for purely temporal clustering, although the proposed procedure can be applied to purely spatial and spatio-temporal cases with minor modifications. When applied to such cases, choosing the correct probability function, *h*(***z***), is critical, and a detailed evaluation along with various simulations is required. Thirdly, our proposed framework is based on the likelihood ratio test statistic, but this is not the only option. For example, the statistical nature of Wald-based and other types of scan statistics have also been studied by other researchers [41, 42]. Some comparison study would be useful for understanding the deviation among those different scan statistics.

Here, it is also worth noting about the account for dependence structures in time series data. Although our likelihood approach assumes a (conditional) independent structure among observations, the approach also shares the notion of partial likelihood [43] that can cope with a dependence structure. It is known that under some reasonable conditions the partial likelihood is able to resemble the amount of information the full joint likelihood possesses. See Kedem and Fokianos [44] for the detail explanation and its theoretical elaboration in time series analysis adopting the GLM framework.

In the spatial context particular, it has been pointed out that the original scan statistic tends to produce more false-positives, when the data contain underlying overdispersion and/or spatial correlation, for which some modification has been proposed in the single-cluster detection test framework [45]. Further investigation on the utility of various multiple-cluster detection test statistics accounting for dependence structures will bring new insights into the future research.

## Conclusion

We have proposed a new testing framework for the simultaneous evaluation of purely temporal multiple-clusters, combining GLM and information criterion approaches that directly stand on the likelihood principle. The framework can, thus, treat the cluster selection problem as the model selection problem and provide a single *p*-value to evaluate the selected multiple clusters as a whole. This feature is unique and beneficial compared to the conventional secondary-cluster procedure that recursively evaluates clusters one by one. Our simulation studies have demonstrated that the new procedure also enables the estimation and evaluation of multiple clusters with high detection power, possessing the consistency property that tends to select the correct number of clusters when the truth is known.
